# Development and validation of a machine learning based early warning scoring system for high altitude polycythemia

**DOI:** 10.3389/fpubh.2025.1739909

**Published:** 2026-01-21

**Authors:** Yangzong Suona, Zhuoga Danzeng, Luobu Gesang, Panduo Zhuoma, Yangjin Baima, Zhuoma Pubu, Wangjie Suolang, Bai Ci, Ju Huang, Quzong Zhaxi, Binyun Liu, Rui Zhang, Quzhen Gesang, Qiangba Dingzeng, Zhuoga Baima

**Affiliations:** 1 High Altitude Medical Research Institute of Tibet Autonomous Region, Lhasa, China; 2 Tibet Autonomous Region People's Hospital, Lhasa, China; 3 Key Laboratory of Transitional Medicine for Human Adaptation to the High Altitude of Tibet Autonomous Region, Tibet Autonomous Region People's Hospital, Lhasa, China; 4 Tibet Autonomous Region Clinical Research Center for High Altitude Diseases, Tibet Autonomous Region People's Hospital, Lhasa, China; 5 Tibet Autonomous Region Center for Disease Control and Prevention, Lhasa, China

**Keywords:** early warning system (EWS), high altitude polycythemia, lifestyle risk factors, machine learning (ML), random forest, XG Boost: eXtreme Gradient Boosting

## Abstract

**Background:**

High-altitude polycythemia (HAPC) lacks a lifestyle-focused risk-stratification tool among lifelong high-altitude residents. Here we aimed to develop and validate a novel machine-learning predictive scoring system for HAPC using readily modifiable lifestyle variables in this population.

**Methods:**

In a high altitude cohort (≥4,500 m, *n* = 1,089), 82 candidate variables were reduced to seven lifestyle predictors via LASSO, Logistic regression, XGBoost and random forest models were trained and compared (10 fold cross validation).

**Results:**

Logistic regression achieved the best balance (AUC 0.848, sensitivity 0.81, specificity 0.79). Low SpO_2_ (< 83%), male sex, age ≥50 year, smoking, hypertension, higher body mass index (BMI) and lower tea consumption were independent predictors.

**Conclusion:**

This score equips frontline health workers in extremely high-altitude, resource-scarce settings to rapidly pinpoint high-risk residents and initiate low-cost lifestyle interventions, thereby curbing the incidence of chronic altitude-related illnesses, easing local medical burdens, and improving overall quality of life for native high-altitude populations.

**Trial Registration:**

ChiCTR2100047945.

## 1 Background

High Altitude Polycythemia (HAPC) is a chronic condition prevalent at high altitudes, characterized by a high incidence rate and significant health risks to affected populations (1, 2). Despite numerous studies exploring the pathogenesis and risk factors of HAPC, the relationship between hypoxic environments and erythrocyte hyperplasia is well-described (1, 2). However, clear guidelines and evidence-based research supporting preventive measures through lifestyle modifications remain unknown (3). Lifestyle interventions have been shown to play a crucial role in preventing and managing chronic diseases (4, 5). Smoking, excessive salt intake, and physical inactivity are key contributors to disease onset and progression (4, 5). The incidence of these diseases and their associated complications can be markedly reduced through health education and lifestyle modifications (5, 6). For instance, health education empowers patients with disease related knowledge, thereby enhancing their health behaviors and facilitating effective management of their conditions. Furthermore, lifestyle interventions yield considerable economic advantages, as the costs associated with prevention are substantially lower than those incurred for treatment (5). Additionally, lifestyle interventions are vital in the prevention and management of erythroplasia. Evidence suggests that modifications such as reducing tobacco use, maintaining balanced work rest schedules, and regulating dietary habits may reduce symptoms of HAPC (7, 8). Nonetheless, there is a relative scarcity of research focused on lifestyle interventions for this condition, and comprehensive early warning and intervention models remain elusive.

In this study, we conducted the first screening of key predictors from 82 epidemiological, physiological and biochemical indicators, which included tea consumption, smoking, gender, blood pressure, age, blood oxygen levels, and body mass index (BMI). We constructed the inaugural HAPC early warning model for high risk populations residing at altitudes exceeding 4,500 meters using machine learning methods, specifically logistic regression, XGBoost, and random forest algorithms. The primary objective of this model is to reduce the prevalence of HAPC through lifestyle interventions and to enhance health awareness within this population via targeted health education, thereby facilitating effective disease prevention. By accurately identifying high risk individuals, we can implement targeted health interventions for lifelong residents of High altitude regions, ultimately strengthening community level disease prevention efforts.

## 2 Methodology

Normality was checked with the Shapiro–Wilk test; non-normal variables were log transformed or handled non-parametrically. Continuous data are presented as mean ± SD or median (IQR); categorical data as counts (%). Group comparisons used *t*-tests/Wilcoxon (continuous) and χ^2^/Fisher tests (categorical); the CMH test was applied for stratified data.

**Figure 1:**
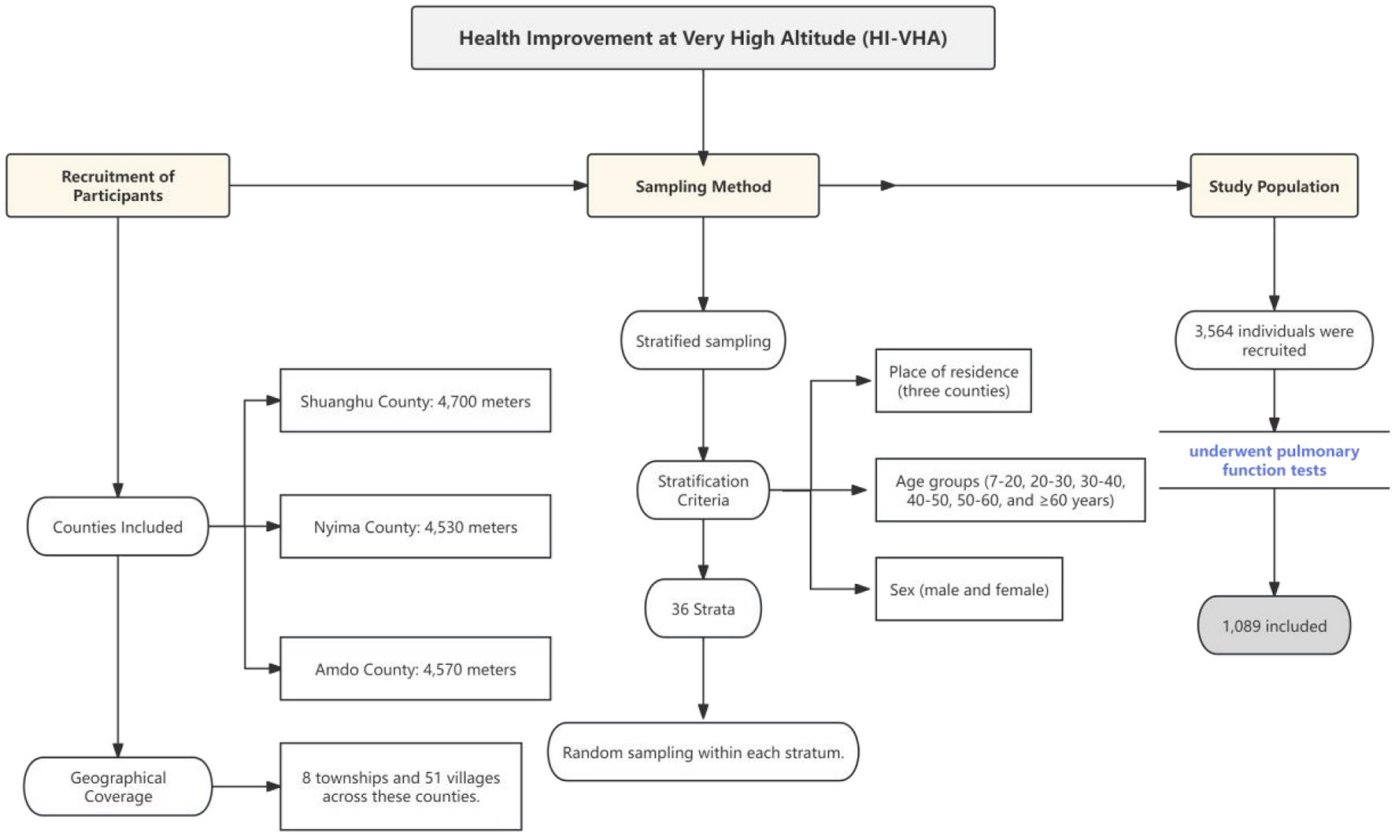
Cohort enrollment flow chart.

From 82 candidate epidemiological, physiological and biochemical variables, LASSO, logistic regression, XGBoost and random forest importance scores were combined to select lifestyle related predictors of HAPC (Figure 1). Logistic regression, XGBoost and random forest models were then built and tuned with 10 fold cross validation. Performance was assessed via AUC, accuracy, sensitivity and specificity, and the best model was adopted for the early warning score. Decision curve analysis (DCA) evaluated net clinical benefit across threshold probabilities. All analyses were performed in R 3.6; *P* ≤ 0.05 was considered significant.

### 2.1 Study population

**Figure 2:**
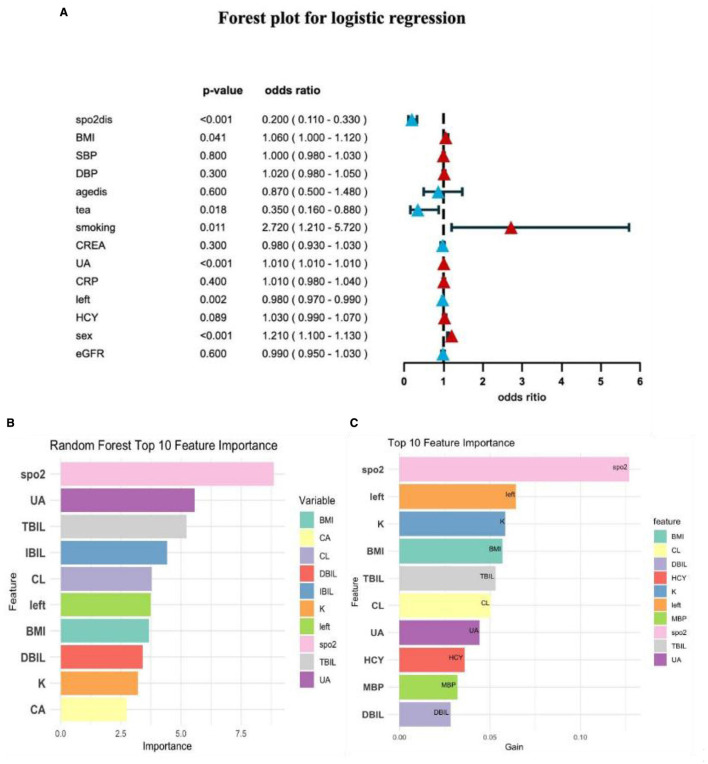
**(A)** Multifactor logistic regression forest plot; **(B)** random forest model variable importance; **(C)** XGBoost variable importance.

On June 24, 2021, we launched the Health Improvement at Very High Altitude (HI VHA) project (ChiCTR2100047945). Through stratified sampling, we recruited 3,564 individuals in the Tibet Autonomous Region (TAR) from three counties: Shuanghu County (4,700 meters above sea level), Nyima County (4,530 meters above sea level), and Amdo County (4,570 meters above sea level). These counties include 8 townships and 51 villages at an altitude of more than 4,500 meters. To ensure representative sampling, the population was categorized into 36 strata based on place of residence (in the three counties), age group (7–20, 20–30, 30–40, 40–50, 50–60, and ≥60 years), and sex (male and female). Researchers randomly sampled within each stratum. After enforcing strict inclusion criteria, a total of 1,089 individuals from HI VHA who received pulmonary function tests were included in this study (Figure 2).

### 2.2 Data collection and processing

The data were sourced from the Health Improvement at Very High Altitude (HI VHA) program. We analyzed 1,089 HI VHA participants (980 non-HAPC, 109 HAPC) with 82 baseline epidemiological, physiological, biochemical and lifestyle variables.

Carbon monoxide exposure was not assessed in the baseline survey and therefore could not be included as a predictor. In addition, although we confirmed whether participants had taken medications at the time of blood sampling to avoid acute drug-related effects on laboratory results, long-term or chronic medication use (e.g., antihypertensive or hypoglycemic agents) was not systematically collected. We acknowledge these limitations and will incorporate detailed CO exposure assessment and comprehensive medication histories in the ongoing follow-up phase of the HI-VHA cohort.

LASSO selected the most predictive features (e.g., tea, smoking, sex, BP, age, SpO_2_). Logistic regression, XGBoost and random forest models were trained and compared; logistic regression (best AUC, sensitivity, and specificity) underpins the final early warning score. A complete list of all 82 variables together with their abbreviations is provided in Supplementary Table S2.

### 2.3 HAPC diagnostic criteria

According to VI World Congress on Mountain Medicine and High Altitude Physiology HAPC was defined as hemoglobin (HGB) levels ≥210 g/L in males and ≥190 g/L in females (9).

### 2.4 Inclusion and exclusion criteria

In this study, pulmonary function testing served as a crucial method for excluding secondary HAPC attributable to COPD, thereby ensuring the accuracy and reliability of the study results while mitigating confounding bias. The results of the pulmonary function tests indicated that the differences in pulmonary function indices between the HAPC group and the non-HAPC group were not significant, further confirming the homogeneity of the study population and providing a robust foundation for subsequent analyses.

Additional definitions included:

- Smoking: current or past smoking history.
- Alcohol consumption: weekly consumption of barley wine, beer, or spirits.
- Tea consumption: regular intake of plain tea, butter tea, or milk tea.
- Body Mass Index (BMI): calculated as weight (kg) divided by height squared (m^2^).
- Waist to Hip Ratio (WHR): waist circumference divided by hip circumference.
- Hypertension: systolic blood pressure (SBP) ≥140 mmHg and/or diastolic blood pressure (DBP) ≥90 mmHg.

(6th edition).

### 2.5 Statistical methodology and model training–evaluation

We conducted all analyses in R 3.6. After confirming normality (Shapiro–Wilk), we split the HI VHA cohort (*n* = 1,089, ≥4,500 m) into 80% training and 20% testing sets. LASSO regression reduced 82 epidemiological, physiological and biochemical variables to seven lifestyle related predictors—tea consumption, smoking, sex, blood pressure, age, SpO_2_ and BMI—which were then fed into logistic regression, XGBoost and random forest models tuned via 10 fold cross validation. Model performance was assessed on the test set using AUC, sensitivity and specificity. Descriptive statistics are presented as mean ± SD or median (IQR) for continuous variables and as counts (%) for categorical variables. Between group differences were evaluated with *t* tests or Wilcoxon rank sum tests for continuous data and χ^2^ or Fisher exact tests for categorical data; stratified comparisons employed the Cochran–Mantel–Haenszel test. All tests were two sided, with *P* ≤ 0.05 considered statistically significant.

### 2.6 Ethics and participant informed consent

The medical research study described in this paper was performed according to the Declaration of Helsinki (https://www.wma.net/what we do/medical ethics/declaration of helsinki/) and approved by the Medical Ethics Committee of Tibet Autonomous Region People's Hospital, with a reference number ID(s): ME TBHP 21 028. Prior to enrollment, all participants provided informed consent by signing the informed consent form.

### 2.7 Questionnaire

The questionnaire includes essential items such as BMI, special diet, records of regularly taken medicines and/or supplements, menstrual status, smoking habits, weekly alcohol consumption (approximate grams of ethanol), and sleeping hours per day. This information will be used to analyze sources of variation in test results and determine the need for a secondary exclusion.

## 3 Result

### 3.1 Clinical characteristics of the population

A total of 1,089 participants were included, comprising 980 non-HAPC and 109 HAPC cases. Compared with the non-HAPC group, HAPC patients had higher hemoglobin levels, were more often male, older, and had lower SpO (Table 1). BMI, SBP, DBP, and mean arterial pressure were significantly elevated. Biochemical differences included higher total bilirubin (TBIL), direct bilirubin (DBIL), indirect bilirubin (IBIL), urea, creatinine, uric acid, triglycerides, low density lipoprotein cholesterol (LDLC), C reactive protein (CRP), potassium, and homocysteine, and lower high density lipoprotein cholesterol (HDLC). Hypertension prevalence was also higher in the HAPC group. Multivariate logistic regression identified SpO_2_ < 83%, male sex, age ≥50 years, smoking, hypertension, higher BMI, and lower tea consumption as independent risk factors for HAPC (Supplementary Table S1).

**Table 1:** Clinical characteristics of patients with HAPC and non-HAPC.

**Characteristic**	**non-HAPC**	**95% CI**	**HAPC**	**95% CI**	***P* value^b^**
	***N*** = **980**^a^		***N*** = **109**^a^		
**Sex**					< 0.001
1	429 (44%)	41%, 47%	70 (64%)	54%, 73%	
2	551 (56%)	53%, 59%	39 (36%)	27%, 46%	
Age	41 (32, 52)	42, 43	46 (36, 55)	44, 49	0.002
agedis	281 (29%)	26%, 32%	45 (41%)	32%, 51%	0.006
**Mothernal**					0.2
0	1 (0.1%)	0.01%, 0.66%	0 (0%)	0.00%, 4.2%	
1	155 (16%)	14%, 18%	17 (16%)	9.6%, 24%	
2	808 (82%)	80%, 85%	87 (80%)	71%, 87%	
3	8 (0.8%)	0.38%, 1.7%	3 (2.8%)	0.71%, 8.4%	
4	8 (0.8%)	0.38%, 1.7%	2 (1.8%)	0.32%, 7.1%	
**Education**					0.4
0	2 (0.2%)	0.04%, 0.82%	0 (0%)	0.00%, 4.2%	
1	15 (1.5%)	0.89%, 2.6%	0 (0%)	0.00%, 4.2%	
2	9 (0.9%)	0.45%, 1.8%	2 (1.8%)	0.32%, 7.1%	
3	189 (19%)	17%, 22%	16 (15%)	8.9%, 23%	
4	765 (78%)	75%, 81%	91 (83%)	75%, 90%	
SpO_2_	83.0 (81.0, 86.0)	83, 83	79.0 (76.0, 82.0)	78, 80	< 0.001
SpO_2_ < 83%	570 (58%)	55%, 61%	24 (22%)	15%, 31%	< 0.001
bpm	80 (70, 89)	79, 81	80 (74, 91)	79, 85	0.2
Height	155 (150, 161)	154, 156	159 (153, 163)	157, 160	< 0.001
Weight	55 (48, 63)	55, 57	63 (57, 74)	62, 67	< 0.001
BMI	22.7 (20.1, 25.7)	23, 24	25.7 (22.6, 29.0)	25, 27	< 0.001
Wrist	78 (69, 87)	77, 79	89 (78, 97)	85, 90	< 0.001
Buds	88 (82, 95)	87, 88	93 (85, 100)	90, 95	< 0.001
WHR	0.89 (0.84, 0.95)	0.89, 0.90	0.94 (0.90, 0.99)	0.93, 0.97	< 0.001
SBP	116 (107, 125)	116, 119	123 (111, 143)	123, 132	< 0.001
DBP	78 (71, 85)	78, 79	83 (75, 95)	83, 89	< 0.001
MBP	91 (83, 97)	91, 92	94 (88, 112)	96, 103	< 0.001
Left	58 (55, 61)	56, 57	58 (53, 62)	45, 54	0.6
Right	58 (55, 60)	55, 57	59 (52, 62)	45, 54	0.4
Smoking	76 (7.8%)	6.2%, 9.7%	11 (10%)	5.4%, 18%	0.4
Drink	64 (6.5%)	5.1%, 8.3%	9 (8.3%)	4.1%, 16%	0.5
Tea	936 (96%)	94%, 97%	98 (90%)	82%, 95%	0.011
Salt intake	120 (90, 150)	130, 145	110 (95, 125)	111, 138	0.2
Families	5.00 (4.00, 6.00)	5.1, 5.4	5.00 (4.00, 7.00)	4.8, 5.6	>0.9
HTN	202 (21%)	18%, 23%	50 (46%)	36%, 56%	< 0.001
Stroke	18 (1.8%)	1.1%, 2.9%	4 (3.7%)	1.2%, 9.7%	0.3
ALT	20 (14, 31)	25, 29	24 (18, 39)	27, 34	< 0.001
AST	22 (18, 27)	24, 26	24 (20, 32)	25, 29	0.002
TP	78.0 (75.1, 81.2)	78, 78	79.0 (76.0, 82.2)	78, 80	0.021
ALB	43.70 (42.00, 45.25)	43, 44	43.30 (42.30, 45.00)	43, 44	0.3
GLO	34.4 (31.9, 36.9)	34, 35	35.3 (33.0, 38.4)	35, 37	0.007
AG	1.30 (1.20, 1.40)	1.3, 1.3	1.20 (1.10, 1.30)	1.2, 1.3	0.012
TBIL	8.5 (6.1, 11.9)	9.2, 9.9	12.1 (8.3, 19.4)	13, 16	< 0.001
DBIL	3.70 (2.50, 5.30)	4.1, 4.5	5.70 (3.30, 7.70)	5.6, 7.2	< 0.001
IBIL	4.7 (2.6, 7.1)	5.2, 5.6	6.7 (4.3, 12.2)	7.4, 9.6	< 0.001
UREA	4.76 (3.94, 5.75)	4.8, 5.0	5.10 (4.20, 6.32)	5.1, 5.8	0.003
CREA	61 (52, 75)	63, 65	69 (55, 80)	66, 72	0.002
UA	353 (285, 433)	358, 370	470 (398, 547)	449, 496	< 0.001
GLU	4.60 (4.30, 5.00)	4.6, 4.7	4.70 (4.20, 5.10)	4.5, 5.1	0.8
TG	0.78 (0.60, 1.02)	0.86, 0.93	0.97 (0.77, 1.24)	0.98, 1.1	< 0.001
TC	4.45 (3.85, 5.10)	4.4, 4.6	4.51 (3.94, 5.22)	4.5, 4.8	0.2
HDLC	1.36 (1.19, 1.58)	1.4, 1.4	1.23 (1.12, 1.40)	1.2, 1.3	< 0.001
LDLC	2.65 (2.09, 3.17)	2.6, 2.7	2.85 (2.34, 3.35)	2.8, 3.1	0.003
CRP	1.20 (0.62, 2.72)	2.4, 3.3	2.38 (1.37, 4.95)	3.2, 5.0	< 0.001
K	4.10 (3.90, 4.40)	4.1, 4.2	4.40 (4.10, 4.70)	4.3, 4.5	< 0.001
NA	141.00 (139.00, 143.00)	141, 141	141.00 (140.00, 142.00)	140, 141	0.4
CL	112.00 (111.00, 114.00)	112, 113	111.00 (109.00, 113.00)	110, 111	< 0.001
CA	2.31 (2.25, 2.37)	2.3, 2.3	2.36 (2.30, 2.44)	2.3, 2.4	< 0.001
MG	0.85 (0.81, 0.89)	0.84, 0.85	0.82 (0.78, 0.87)	0.80, 0.84	< 0.001
Pi	1.12 (1.01, 1.25)	1.1, 1.1	1.12 (0.97, 1.25)	1.1, 1.2	0.4
HCY	16.5 (13.5, 20.7)	17, 18	19.6 (16.5, 24.3)	20, 22	< 0.001
eGFR	152 (145, 165)	152, 154	149 (141, 159)	145, 151	0.002
WBC	6.30 (5.40, 7.50)	6.4, 6.6	6.30 (5.20, 7.10)	6.0, 6.6	0.4
LYMPH	2.00 (1.70, 2.30)	2.0, 2.1	2.10 (1.60, 2.40)	1.9, 2.1	0.9
Middle cells	0.40 (0.30, 0.50)	0.41, 0.43	0.30 (0.30, 0.50)	0.35, 0.41	0.011
NEUT	3.80 (3.00, 4.80)	4.0, 4.1	3.80 (3.20, 4.40)	3.7, 4.2	0.7
LYMPHP	32 (27, 38)	32, 33	33 (29, 37)	31, 34	0.9
Middle cells%	6.60 (5.30, 8.00)	6.6, 6.9	6.00 (5.00, 7.80)	6.0, 6.7	0.058
NEUTP	61 (55, 67)	60, 61	61 (56, 66)	60, 63	0.6
VCMAX	3.60 (3.07, 4.26)	3.6, 3.7	3.74 (3.09, 4.44)	3.6, 4.0	0.3
**Pulmonary function**					
ERV	1.29 (0.88, 1.79)	1.3, 1.4	1.25 (0.86, 1.94)	1.3, 1.6	0.6
IC	2.29 (1.84, 2.75)	2.3, 2.3	2.24 (1.91, 2.64)	2.2, 2.5	>0.9
MV	24 (18, 30)	24, 25	24 (19, 31)	24, 27	0.2
VT	1.19 (0.91, 1.51)	1.2, 1.3	1.19 (0.98, 1.49)	1.2, 1.3	0.7
FVCEX	3.53 (3.02, 4.15)	3.5, 3.6	3.64 (2.98, 4.35)	3.5, 3.9	0.3
FEV1	2.98 (2.56, 3.57)	3.0, 3.1	3.16 (2.60, 3.74)	3.0, 3.3	0.2
FEV1FVCEX	85 (81, 90)	85, 86	86 (82, 90)	85, 87	0.2
PEF	5.93 (4.57, 7.54)	6.0, 6.3	6.18 (4.79, 7.86)	6.1, 7.0	0.11
MEF75	5.59 (4.24, 7.15)	5.7, 5.9	5.91 (4.54, 7.41)	5.8, 6.6	0.079
MEF50	4.26 (3.22, 5.40)	4.3, 4.5	4.33 (3.36, 5.46)	4.2, 4.8	0.4
MEF25	1.70 (1.21, 2.27)	1.8, 1.9	1.78 (1.33, 2.27)	1.7, 2.0	0.4
MEF2575	3.53 (2.71, 4.43)	3.6, 3.7	3.62 (2.81, 4.64)	3.5, 4.0	0.3
**FEV135**					
VEXT	0.16 (0.12, 0.23)	0.18, 0.19	0.16 (0.11, 0.22)	0.15, 0.18	0.14
MIF50MEF50	117 (93, 145)	122, 128	120 (97, 147)	120, 142	0.4
FEV2	110 (99, 121)	109, 112	111 (101, 125)	110, 120	0.14
FEV1FVCEX1	102 (96, 107)	101, 102	102 (98, 108)	101, 104	0.3

^a^Median (Q1, Q3); n (%).

CI, confidence interval.

^b^Wilcoxon rank sum test; Pearson's Chi squared test; Fisher's exact test.

Regarding laboratory indices, Table 1 shows the HAPC group exhibited significant abnormalities in several biochemical parameters, including elevated levels of total bilirubin (TBIL), direct bilirubin (DBIL), indirect bilirubin (IBIL), urea (UREA), creatinine (CREA), uric acid (UA), triglycerides (TG), low density lipoprotein cholesterol (LDLC), and C reactive protein (CRP), while high density lipoprotein cholesterol (HDLC) levels were notably lower (*P* < 0.001). Additionally, serum potassium (K) levels were significantly higher in the HAPC group compared to the Non-HAPC group (*P* < 0.001), whereas the differences in chloride (CL), calcium (CA), magnesium (MG), and phosphorus (Pi) levels were not significant (*P* > 0.05). Homocysteine (HCY) levels were also significantly elevated in the HAPC group relative to the Non-HAPC group (*P* < 0.001), and the estimated glomerular filtration rate (eGFR) was slightly lower than that of the Non-HAPC group (*P* = 0.002). In terms of lung function indices, the HAPC group showed no significant differences in maximal ventilation (VCMAX), residual airway volume (ERV), inspiratory capacity (IC), minute ventilation (MV), tidal volume (VT), expiratory volume with exertion (FVCEX, FEV1, FEV1/FVCEX), peak expiratory flow rate (PEF), and maximal expiratory flow rate at mid expiratory (MEF75, MEF50, MEF25, MEF25–75) compared to the Non-HAPC group (*P* > 0.05). However, some measures, such as FEV1/FVCEX and PEF, were slightly decreased in the HAPC group (*P* > 0.05). These results indicate that patients with HAPC exhibit significant differences in various clinical features, biochemical indices, and pulmonary function indices compared to non-patients, which may be closely associated with the onset and progression of the disease.

## 4 Predictors selection

In this study, we identified the top ten variables that significantly contributed to the predictive ability of the model by integrating feature importance scores from two machine learning algorithms: Random Forest (Figure 2B) and XGBoost (Figure 2C). The selected variables included oxygen saturation (SpO_2_), uric acid (UA), total bilirubin (TBIL), indirect bilirubin (IBIL), chlorine (CL), left cerebral oxygen saturation (LEFT), body mass index (BMI), direct bilirubin (DBIL), potassium (K), and calcium (CA). We particularly focused on factors closely associated with lifestyle, such as tea consumption, smoking, gender, blood pressure, age, oxygen saturation, and BMI.

Multivariate logistic regression analysis revealed that SpO_2_ (blood oxygen saturation) exhibited a highly significant effect in both algorithms, indicating that blood oxygen levels play a crucial role in predicting the target variable. Additionally, BMI (body mass index), which reflects an individual's weight to height ratio, ranked prominently in both algorithms, further emphasizing the importance of weight management in health prediction. Male sex emerged as a significant risk factor (*P* < 0.001) in the logistic regression analysis. Lifestyle factors, including tea consumption and smoking, demonstrated significant predictive power in the logistic regression forest plot, with tea consumption showing a significant negative association with the predicted outcomes (dominance ratio = 0.350, 95% confidence interval: 0.160 0.880, *P* = 0.018), while smoking exhibited a significant positive association (dominance ratio = 2.720, 95% confidence interval: 1.210 5.720, *P* = 0.011).

In summary, we ultimately selected a set of lifestyle based predictors through a comprehensive analysis of feature importance. This analysis involved utilizing random forest feature importance and examining logistic regression forest plots. The selected factors included tea consumption, smoking status, gender, blood pressure, age, oxygen saturation, and body mass index (BMI). Each of these factors exhibited significant predictive capability within the model, thereby establishing a crucial foundation for subsequent modeling efforts.

## 5 Model validation

**Figure 3:**
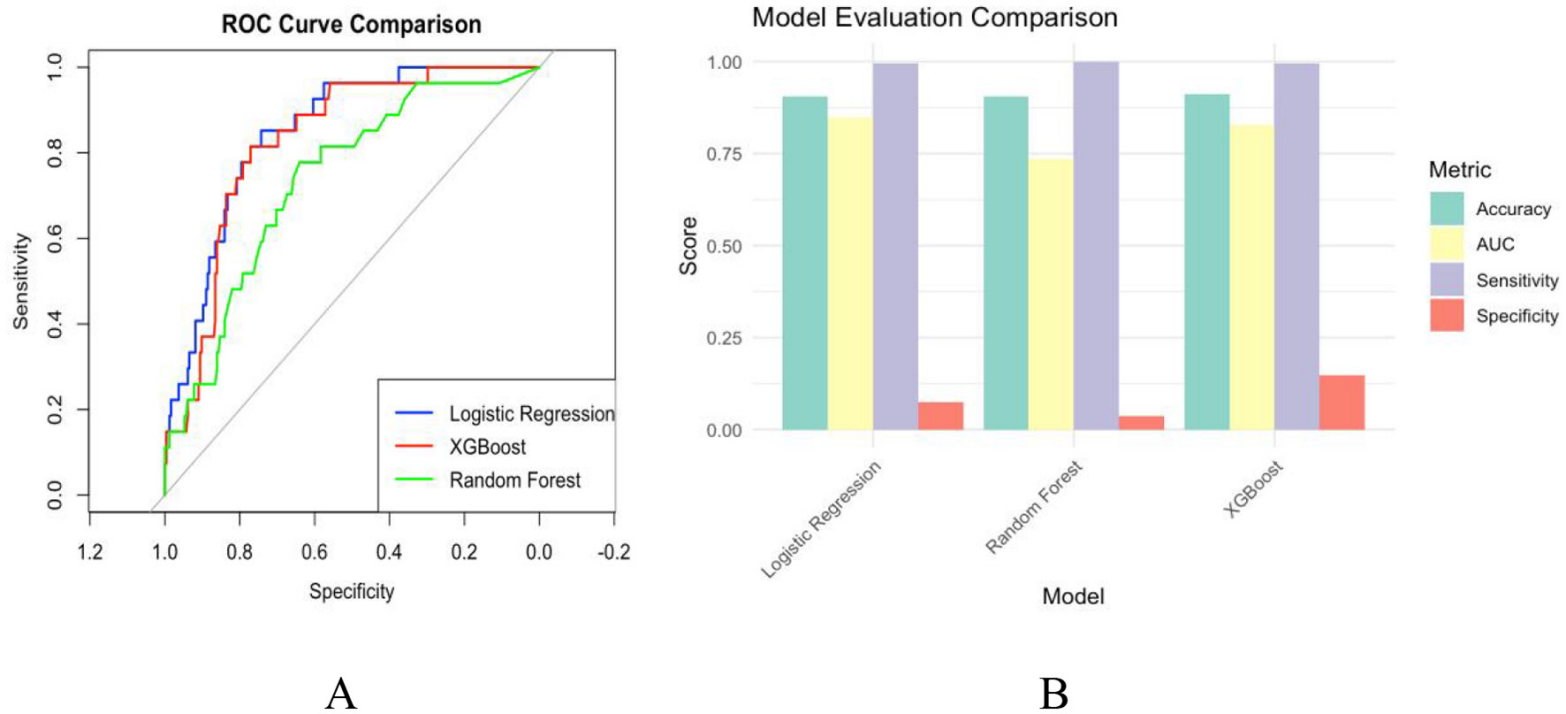
**(A)** Blue for logistic regression, Red for XGBoost, and Green for Random Forest. **(B)** teal for accuracy, yellow for AUC, purple for sensitivity, and red for specificity.

In the model evaluation, we compared the performance of three models: logistic regression, XGBoost, and random forest. The results are presented in Figures 3A, B. Figure 2A illustrates the scores of the three models across four metrics: Accuracy, Area Under the Curve (AUC), Sensitivity, and Specificity. The performance of these models on these indicators is relatively close, though slight differences are evident in certain aspects. The Random Forest model exhibits marginally higher accuracy and AUC compared to the other two models, indicating its superior ability to differentiate between positive and negative samples. Regarding sensitivity, the performance of all three models is nearly identical, suggesting they are comparable in recognizing positive samples. In terms of specificity, the XGBoost model slightly outperforms the others, demonstrating its advantage in identifying negative samples.

Figure 2A further illustrates the Receiver Operating Characteristic (ROC) curves of the three models, evaluating their performance by plotting sensitivity and specificity at various thresholds. The closer the ROC curve is to the upper left corner, the better the model's performance. In Figure 2A, the ROC curves for both the XGBoost and Random Forest models are closer together and significantly outperform the logistic regression model, which is consistent with the Area Under the Curve (AUC) evaluation presented in the same figure.

This study evaluated the clinical utility of three distinct predictive models: random forest (rf_model), XGBoost (xgb_model), and logistic regression (logistics_model), across various threshold probabilities using decision curve analysis (DCA). As illustrated in Figure 3A, the random forest model (rf_model) demonstrated significant net benefits at lower threshold probabilities (0.0–0.2), which progressively diminished with increasing threshold probabilities. Nevertheless, across most threshold ranges, the net gain of the model surpassed that of both the “all or nothing” (red curve) and “all or nothing” (green curve) strategies, suggesting superior potential for clinical applications. Figure 3B presents the decision curve for the XGBoost model (xgb_model). Consistent with the random forest model, the XGBoost model also exhibited substantial net gains at lower threshold probabilities. In the majority of threshold ranges, the net benefit of the XGBoost model also exceeded that of the “all or nothing” strategies, indicating its considerable value in clinical decision making.

**Figure 4:**
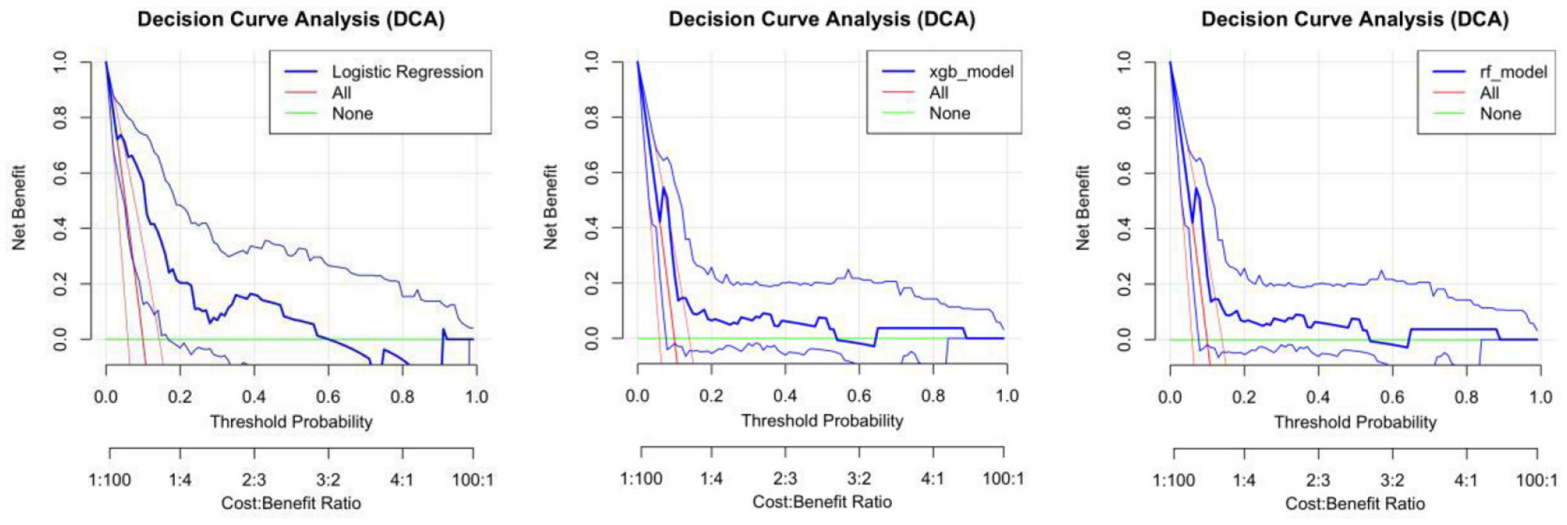
DCA curve **(A)** logistic regression model **(B)** random forest model **(C)** XGBoost model.

Figures 4A–C illustrates the decision curve of the logistic regression model (lr_model). Compared to the first two models, the logistic regression model shows slightly lower net benefits at certain threshold probabilities. Nevertheless, within the lower threshold probability range, the net return of this model remains higher than that of the “all or nothing” strategy, indicating its potential clinical applicability. Analyzing the decision curves of the three models (Figure 3), it is evident that in the lower threshold probability range, both the random forest and XGBoost models demonstrate higher net benefits than the logistic regression model, suggesting superior clinical utility. However, in the higher threshold probability range, the net returns of all three models are relatively similar and fall below those of the “do it all” strategy.

Model evaluation ranked SpO_2_ as the dominant predictor, followed by DBP, sex, BMI, SBP, smoking and tea (Supplementary Figure S1). SHAP analysis showed that higher SpO_2_ and BMI positively shifted predictions, whereas elevated DBP and SBP had negative effects (Supplementary Figure S2A). At the individual level, SpO_2_ = 79 increased the predicted value by 1.47, while female sex reduced it by 0.567 (Supplementary Figures S2B, C and Figure 4).

## 6 Discussion

HAPC is a common, under recognized chronic disease that markedly impairs health and quality of life in high altitude populations. Although its pathogenesis and risk factors are increasingly understood, evidence based lifestyle interventions and guidelines remain scarce. Because preventive lifestyle measures are cost effective and curb both incidence and complications, we built a machine learning early warning model for HAPC that pinpoints key lifestyle predictors. The resulting simple, efficient tool equips public health workers to quickly identify high risk individuals and initiate early intervention, filling a critical gap in high altitude health management.

### 6.1 Key predictors

Blood oxygen saturation (SpO_2_) is a critical indicator of the physiological status in high-altitude environments and plays a central role in the development of HAPC (1, 10, 11). In this study, low oxygen saturation (SpO_2_ < 83%) at altitudes above 4,500 meters emerged as a strong predictor of HAPC (OR = 4.35, 95% CI: 3.33–5.88). Hypoxic conditions characteristic of highland regions may exacerbate erythrocyte hyperplasia, ultimately contributing to HAPC. Consistent with previous literature, this finding highlights the importance of routine SpO_2_ monitoring in high-altitude populations and suggests that improving oxygenation (through oxygen therapy or environmental modifications) may help reduce HAPC risk (12, 13).

Regarding tea consumption, we observed a significant negative association with HAPC (OR = 0.47, 95% CI: 0.26–0.85). Although direct evidence linking tea intake to HAPC prevention is lacking, existing studies suggests that tea may alleviate high-altitude-related symptoms by modulating blood viscosity and improving microcirculation. A study published in the European Journal of Applied Physiology, further showed that tea reduce fatigue at high altitude, implying potential physiological and psychological benefits for adaptation (14). These observations provide a valuable reference for lifestyle interventions among highlanders.

Smoking history was also associated with increased HAPC risk (OR = 2.72, 95% CI: 1.21–5.72). This relationship may stem from smoking-induced reductions in oxygenation capacity and increased blood viscosity. Not only does smoking diminish oxygen saturation, but it may also exacerbate the HAPC symptoms by heightening inflammatory responses and oxidative stress (15, 16). Therefore, smoking cessation should be considered a key component of lifestyle-based prevention.

Male sex was identified as a risk factor (OR = 1.96, 95% CI: 1.12 1.96). This may related to physiological and hormonal differences, as men typically demonstrate higher erythropoietin levels and greater erythropoietic capacity, potentially promoting erythrocyte proliferation under hypoxic conditions. Some studies also suggest that men may have lower physiological adaptability to high altitude than women, further increasing susceptibility (17, 18).

Body Mass Index (BMI) and Blood Pressure were significantly associated with HAPC (2). High BMI (OR = 3.06, 95% CI: 1.55 5.98) and hypertension (OR = 1.58, 95% CI: 1.11 2.24) were identified as independent risk factors. Specifically, elevated systolic (OR = 1.02, 95% CI: 1.011 1.029) and diastolic blood pressure (OR = 1.58, 95% CI: 1.11 2.24) contributed to increased HAPC risk. Hypertension and obesity may promote HAPC through effects on metabolism and blood rheology, thereby increasing erythropoiesis and blood viscosity (2, 18). Maintaining a healthy body weight and optimal blood pressure is therefore essential for the preventing HAPC.

Age ≥50 years also showed a significant association with HAPC (OR = 1.97, 95% CI: 1.55 2.50). Declining physiological adaptability with age may increase vulnerability to chronic hypoxia leading to enhanced erythrocyte proliferation (2, 16, 19).

## 7 Conclusion

We developed a concise early warning score for high altitude erythropoietic cellular disorder (HAPC) using logistic regression, XGBoost and random forest. Logistic regression proved optimal (AUC = 0.848), highlighting seven lifestyle related predictors: tea intake, smoking, sex, blood pressure, age, SpO_2_ and BMI. The resulting tool enables front line health workers to rapidly flag high risk residents and initiate targeted lifestyle interventions—smoking cessation, weight control and improved oxygenation—potentially cutting HAPC incidence and improving high altitude health.

### 7.1 Limitations and future directions

Our study has several limitations. First, the diagnostic criterion for HAPC was based on hemoglobin cut-offs rather than bone-marrow or JAK2 testing; however, the prevalence of PV in native Tibetans is extremely low, and sensitivity analyses using 200/180 g/L thresholds did not alter the top predictors (Supplementary Figure S5). Second, lifestyle variables were self-reported, yet a pilot pictorial diary showed good reliability (ICC 0.78). To avoid missing potentially informative predictors at this exploratory stage, we initially included a broad range of biochemical, physiological, and lifestyle indicators, even though some biochemical measures are not direct causal drivers of polycythemia. After integrating multiple feature-selection methods, the final model relies mainly on easily obtainable lifestyle-related variables, which is consistent with our aim of developing a practical tool for frontline use. In addition, follow-up of the HI-VHA cohort is ongoing, and the longitudinal data will enable temporal validation to further assess the model's stability and clinical applicability. Third, external validation is ongoing in a 1,200 m cohort; here we provide internal–external validation by iteratively withholding one county (pooled AUC 0.834). In addition, follow-up of the HI-VHA cohort is still in progress. Once the longitudinal follow-up data are complete, we plan to conduct temporal validation within the cohort to further evaluate the stability and clinical applicability of the model. Finally, the score predicts risk but does not prove that lifestyle change reduces Hb; a cluster-RCT (NCT05984234) using this tool is underway and will report 12-month outcomes in 2026.

## Data Availability

The raw data supporting the conclusions of this article will be made available by the authors, without undue reservation.
