# Supplementary figures and images for: Development and validation of a machine learning based early warning scoring system for high altitude polycythemia
Source: Front Public Health. 2026 Jan 21;13:1739909. doi: 10.3389/fpubh.2025.1739909 (PMC12868181; doi:10.3389/fpubh.2025.1739909)

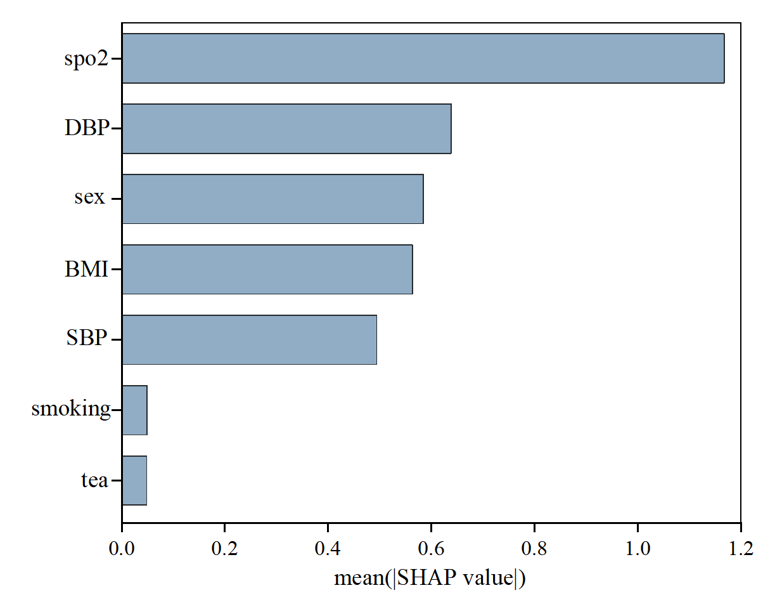

Supplement: Supplementary file 1 [file Supplementary_file_1.tif]

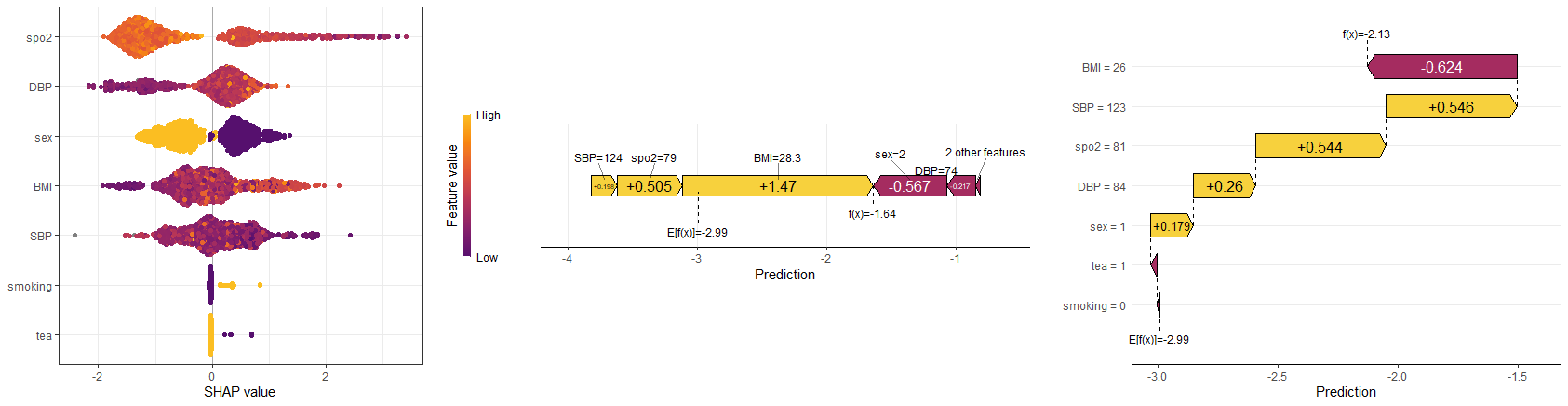

Supplement: Supplementary file 2 [file Supplementary_file_2.tif]
