# Supplementary material for: Development and validation of a machine learning based early warning scoring system for high altitude polycythemia
Source: Front Public Health. 2026 Jan 21;13:1739909. doi: 10.3389/fpubh.2025.1739909 (PMC12868181; doi:10.3389/fpubh.2025.1739909)
Supplement: Supplementary file 3 [file Table_1.docx]

**Supplementary** **Table 1 univariate analysis and mulvariate analysis**

|  | **univariate analysis** | | |  | **mulvariate analysis** | | |
| --- | --- | --- | --- | --- | --- | --- | --- |
| **variable** | OR | 95%CI | | **variable** | OR | 95%CI | |
|  |  | Lower limit | upper limit |  |  | Lower limit | upper limit |
| **SaO2<83%** | 3.7 | 2.94 | 4.76 | **SaO2<83%** | 4.35 | 3.33 | 5.88 |
| **male** | 1.96 | 1.55 | 2.49 | **male** | 1.96 | 1.12 | 1.96 |
| **age≥50** | 1.97 | 1.55 | 2.5 | **age≥50** | 1.33 | 1.01 | 1.76 |
| **WHR** | 353.067 | 63.206 | 1972.227 | **WHR** | 11.62 | 0.92 | 146.04 |
| **MBP** | 1.02 | 1.011 | 1.029 | **MBP** |  |  |  |
| **Cerebral oxygen saturation left** | 1.056 | 1.031 | 1.082 | **Cerebral oxygen saturation left** | 1 | 0.98 | 1.01 |
| **smoking** | 1.55 | 1.037 | 2.317 | **smoking** | 2.25 | 1.31 | 3.89 |
| **tea** | 0.455 | 0.301 | 0.689 | **tea** | 0.47 | 0.26 | 0.85 |
| **HTN** | 2.039 | 1.557 | 2.67 | **HTN** | 1.58 | 1.11 | 2.24 |
| **CREA** | 1.012 | 1.003 | 1.021 | **CREA** | 0.95 | 0.93 | 0.98 |
| **UA** | 1.008 | 1.007 | 1.009 | **UA** | 1.01 | 1.01 | 1.01 |
| **CRP** | 1.02 | 1.003 | 1.036 | **CRP** | 1 | 0.97 | 1.03 |
| **HCY** | 1.037 | 1.023 | 1.052 | **HCY** | 1.02 | 1 | 1.05 |
| **eGFR** | 0.991 | 0.984 | 0.998 | **eGFR** | 0.98 | 0.96 | 1 |
| **Middle cells** | 0.456 | 0.196 | 1.062 |  |  |  |  |
| **Middle cells%** | 0.874 | 0.814 | 0.938 | **Middle cells%** | 1.01 | 0.99 | 1.03 |
