# Supplementary material for: Development and validation of a machine learning based early warning scoring system for high altitude polycythemia
Source: Front Public Health. 2026 Jan 21;13:1739909. doi: 10.3389/fpubh.2025.1739909 (PMC12868181; doi:10.3389/fpubh.2025.1739909)
Supplement: Supplementary file 4 [file Table_2.docx]

**Supplementary** **Table 2 A** complete abbreviations list of all 82 variables

| Variable | Abbreviation | Unit | Category |
| --- | --- | --- | --- |
| Age |  | years | Demographic |
| Sex |  |  | Demographic |
| Height |  | cm | Anthropometric |
| Weight |  | kg | Anthropometric |
| Body mass index | BMI | kg/m² | Anthropometric |
| Waist–hip ratio | WHR |  | Anthropometric |
| Systolic blood pressure | SBP | mmHg | Physiological |
| Diastolic blood pressure | DBP | mmHg | Physiological |
| Heart rate | HR | bpm | Physiological |
| Oxygen saturation | SpO₂ | % | Physiological |
| Left cerebral oxygen saturation | LEFT | % | Physiological |
| Smoking history |  |  | Lifestyle |
| Tea consumption |  |  | Lifestyle |
| Alcohol consumption |  |  | Lifestyle |
| Sleep duration |  | hours | Lifestyle |
| Total bilirubin | TBIL | μmol/L | Biochemical |
| Direct bilirubin | DBIL | μmol/L | Biochemical |
| Indirect bilirubin | IBIL | μmol/L | Biochemical |
| Uric acid | UA | μmol/L | Biochemical |
| Sodium | Na | mmol/L | Biochemical |
| Potassium | K | mmol/L | Biochemical |
| Chloride | CL | mmol/L | Biochemical |
| Calcium | CA | mmol/L | Biochemical |
| Creatinine | Cr | μmol/L | Biochemical |
| Blood urea nitrogen | BUN | mmol/L | Biochemical |
| ALT | ALT | U/L | Biochemical |
| AST | AST | U/L | Biochemical |
| Albumin | ALB | g/L | Biochemical |
| Globulin | GLB | g/L | Biochemical |
| LDL cholesterol | LDL-C | mmol/L | Biochemical |
| HDL cholesterol | HDL-C | mmol/L | Biochemical |
| Total cholesterol | TC | mmol/L | Biochemical |
| Triglycerides | TG | mmol/L | Biochemical |
| Pulmonary function indices |  |  |  |
| Maximum vital capacity | VCMAX | L | Pulmonary function |
| Expiratory reserve volume | ERV | L | Pulmonary function |
| Inspiratory capacity | IC | L | Pulmonary function |
| Minute ventilation | MV | L/min | Pulmonary function |
| Tidal volume | VT | L | Pulmonary function |
| Forced vital capacity (exhalation) | FVCEX | L | Pulmonary function |
| Forced expiratory volume in 1 second | FEV1 | L | Pulmonary function |
| FEV1 / FVC ratio | FEV1/FVCEX |  | Pulmonary function |
| Forced expiratory volume in 2 seconds | FEV2 | L | Pulmonary function |
| Peak expiratory flow | PEF | L/s | Pulmonary function |
| Maximum expiratory flow at 75% | MEF75 | L/s | Pulmonary function |
| Maximum expiratory flow at 50% | MEF50 | L/s | Pulmonary function |
| Maximum expiratory flow at 25% | MEF25 | L/s | Pulmonary function |
| Maximum mid-expiratory flow | MEF25–75 | L/s | Pulmonary function |
| Forced expiratory volume at 35% | FEV135 | L/s | Pulmonary function |
| Expiratory ventilation | VEXT | L/min | Pulmonary function |
| MIF50/MEF50 ratio | MIF50MEF50 |  | Pulmonary function |
| FEV1/FVC (first second) | FEV1FVCEX1 |  | Pulmonary function |
